# Tracking and evaluating motion skills in laparoscopy with inertial sensors

**DOI:** 10.1007/s00464-023-09983-y

**Published:** 2023-03-28

**Authors:** Christian Heiliger, Dorian Andrade, Christian Geister, Alexander Winkler, Khaled Ahmed, Alessandra Deodati, Viktor H. Ehrlich v. Treuenstätt, Jens Werner, Andreas Eursch, Konrad Karcz, Alexander Frank

**Affiliations:** 1 Department of General, Visceral, and Transplant Surgery, Ludwig-Maximilians-University (LMU) Hospital, 81377 Munich, Germany; 2 Department of Mechanical, Automotive and Aeronautical Engineering, University of Applied Sciences, Munich, Germany; 3 Chair for Computer Aided Medical Procedures & Augmented Reality (CAMP), Technical University of Munich (TUM), Munich, Germany

**Keywords:** Inertial sensors, Laparoscopy, Motion tracking, Surgical training

## Abstract

**Background:**

Analysis of surgical instrument motion is applicable in surgical skill assessment and monitoring of the learning progress in laparoscopy. Current commercial instrument tracking technology (optical or electromagnetic) has specific limitations and is expensive. Therefore, in this study, we apply inexpensive, off-the-shelf inertial sensors to track laparoscopic instruments in a training scenario.

**Methods:**

We calibrated two laparoscopic instruments to the inertial sensor and investigated its accuracy on a 3D-printed phantom. In a user study during a one-week laparoscopy training course with medical students and physicians, we then documented and compared the training effect in laparoscopic tasks on a commercially available laparoscopy trainer (Laparo Analytic, Laparo Medical Simulators, Wilcza, Poland) and the newly developed tracking setup.

**Results:**

Eighteen participants (twelve medical students and six physicians) participated in the study. The student subgroup showed significantly poorer results for the count of swings (CS) and count of rotations (CR) at the beginning of the training compared to the physician subgroup (*p* = 0.012 and *p* = 0.042). After training, the student subgroup showed significant improvements in the rotatory angle sum, CS, and CR (*p* = 0.025, *p* = 0.004 and *p* = 0.024). After training, there were no significant differences between medical students and physicians. There was a strong correlation between the measured learning success (LS) from the data of our inertial measurement unit system (LS_IMU_) and the Laparo Analytic (LS_Lap_) (Pearson’s *r* = 0.79).

**Conclusion:**

In the current study, we observed a good and valid performance of inertial measurement units as a possible tool for instrument tracking and surgical skill assessment. Moreover, we conclude that the sensor can meaningfully examine the learning progress of medical students in an ex-vivo setting.

The laparoscopic approach is preferred for several abdominal surgeries as it involves reduced postoperative pain, shorter hospital stays, and a lower rate of complications than open surgery. However, laparoscopic surgery requires much experience to handle the instruments skillfully. The learning curve is steep, and despite the many training systems that are around, many novice surgeons acquire the required skills usually through active participation in minimally invasive surgeries (MIS) with the "See one, Do one, Teach one” approach [1].

Instead, through surgical training in a safe and controlled environment outside the operating room (OR), novice surgeons can gain substantial laparoscopic psychomotor coordination, and their learning success can be monitored. In this regard, many determinant factors for efficient handling of instruments were based on a bundle of motion-based parameters and time, furtherly force-based parameters were also considered as a reflection of how tissue-traumatizing would be the manipulation [2, 3].

There are two types of ex-vivo training systems: box trainers and virtual reality trainers. Both can increase laparoscopic skills and monitor the learning curve through motion-tracking analysis [2–6]. Several surgical navigation systems that use precise instruments and patient tracking are globally found in many ORs, e.g., combined with intraoperative CT, MRI or X-ray imaging used in neuro- or orthopedic surgery. These systems can accurately determine the poses of active or passive tracking targets. Optical tracking systems have high accuracy and can cover a sufficiently large tracking volume. They have the inherent problem of relying on an uninterrupted line of sight to the area they monitor, which may be lost during the surgical workflow. Markerless optical systems may be possible; however, they exhibit not enough accuracy for surgical applications [7].

Magnetic tracking systems, as an alternative, do not suffer from line-of-sight issues but exhibit relatively lower accuracy and are challenging to apply in the OR due to interference with electronic devices and metallic instruments in the magnetic field [8].

Ren et al. investigated the integration of inertial sensors with either of the existing technologies to increase the sensor's accuracy and compensate for the line-of-sight potential error, which was less feasible to be applied in OR setting [9].

Another publication by Horeman et al. introduced the development of a trainer that can measure task time, force and motion data for multiple port and single port laparoscopy. They included some sensors inside the box, so there is a need to perform the training within the whole system [10].

Computer vision methods can also enable good skill assessment and recording of learning success at a low cost. However, as an indirect recording of movement, rotation of the instrument, movements outside the camera's field of view, and, in particular, instrument movements and interactions outside the situs with the OR nursing staff cannot be recorded [11].

An inertial navigation system is a spatial combination of multiple sensors. Common combinations consist of accelerometers, angular rate sensors, and magnetometers. From their data, the orientation and position of an object in space can be derived. They offer a promising small-sized possibility for tracking with high suitability in MIS, despite their relatively low accuracy. As a small wireless device, an inertial sensor, can be suitable for OR use, as it can be covered by a sterilizable cover [12].

The current study not only developed and validated a digital instrument tracking system but also analyzed its potential uses in monitoring and assessing the performance of laparoscopic surgeons.

## Materials and methods

### Sensor implementation

Laparoscopic instruments are limited to four degrees of freedom once inserted into a trocar: only one translation in depth (surge) and three rotations (pitch *ψ* (up and down), yaw *θ* (left and right), roll *φ* (along and instrument axis)) around the *x*, *y*, and *z* axes.

We chose the TDK SmartBug (MD-42688-P, TDK InvenSense, San Jose, USA) inertial sensor to track the motion of the laparoscopic instruments. This sensor includes calibration, data processing, and a data recording function at an economical price of ca. 80€ per device. The sensor consisted of a 3-axis accelerometer, 3-axis gyroscope, and 3-axis magnetometer.

The measurement error increased over time due to scattering and systematic deviations. Therefore, the emitted data from the multiple sensor components were fused and treated by a Kalman-filter to determine the orientation of the instrument. The sensor transmitted the data with a sampling rate of 100 Hz over a Bluetooth connection to a Windows 10 (Microsoft, Redmond, USA) desktop computer, which then processed the data in MATLAB (MathWorks, Natick, USA). The sampling rate in addition with the sensors sensitivity is suitable for tracking micro- as well as macro-movements.

### Calibration and accuracy evaluation on a phantom

**Fig. 1:**
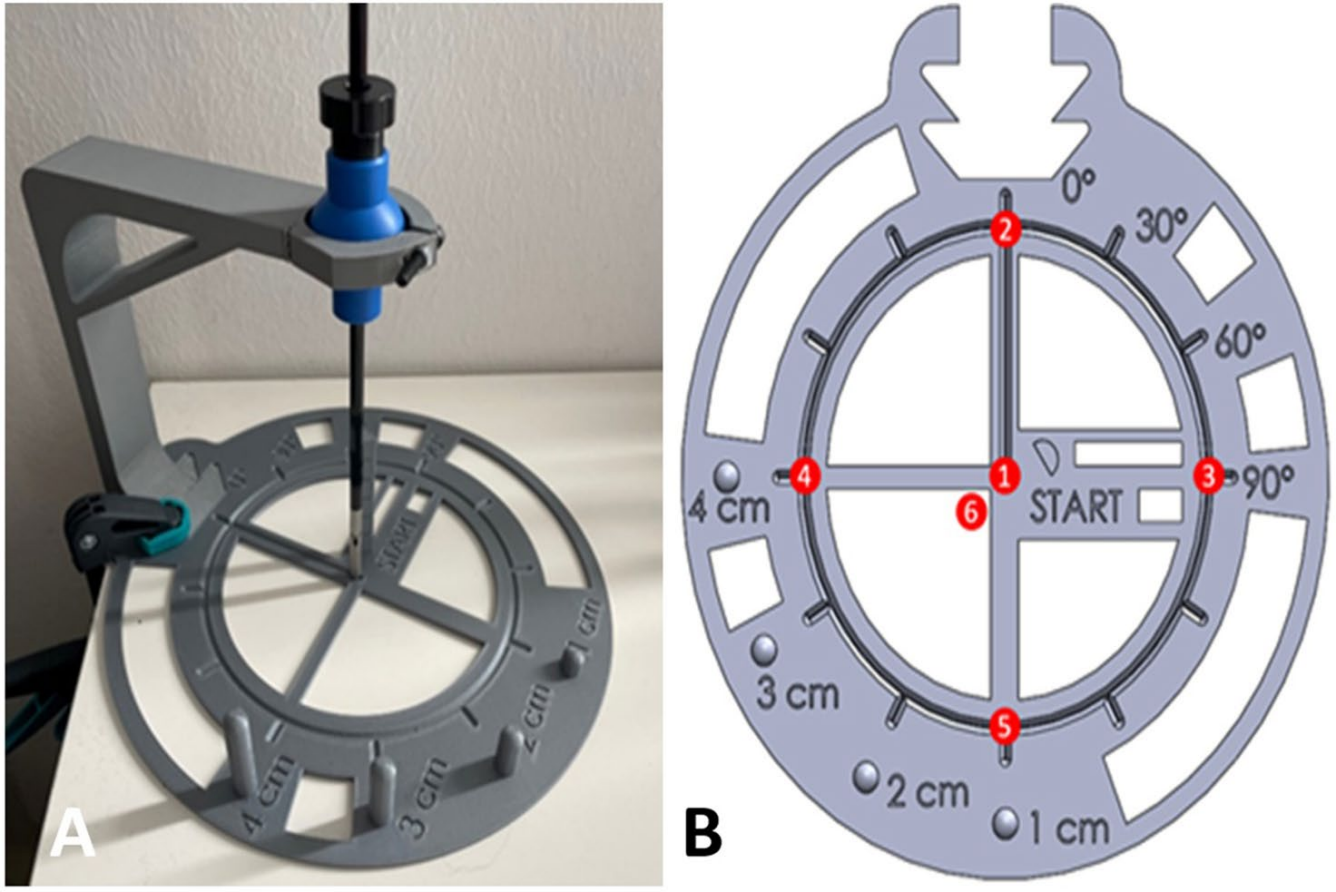
**A** and **B** The positions of different landmarks are known in reference to the position of a surgical instrument. The instrument is held in a trocar-like situation. The instrument tip is steered to the landmarks. The calculated values for the position can be compared with the landmark’s coordinates. Values for the orientation and position were calculated separately

We evaluated the accuracy of the measurement setup on a 3d-printed phantom. The measurement phantom consisting of a mount for the instrument and a flat plate was 3d printed in Acrylonitrile Butadiene Styrene (ABS), a rigid plastic. The instrument was restricted in its translational movement, like the bearing in a trocar. The measurement phantom consists of a central starting point and six reference points. The instrument tip is moved from the center to one reference point in ten repetitions. Each position is being hold for approximately three seconds, while each position change takes one second. The procedure is repeated for the six reference points. The measured orientation was derived by the average measurement of one position. For each of these positions, the measured orientation was compared to the actual value from the geometry of the 3D model (Fig. 1).

The rotation measurements (mean, standard deviation, and difference to the actual value) from the phantom evaluation were recorded (Table 1). Accuracy overall was satisfactory for the intended use, with the highest absolute mean error for one target in one axis (pitch) of 6.40°. The highest standard deviation for one axis was 1.15° (also pitch). Pitch (*ψ*) was overall less precise because the axial orientation was done manually. In contrast, roll (*ϕ*) and yaw (*θ*) were defined by stops and, therefore, less prone to error.

**Table 1:** Mean and Standard Deviation of rotational movements (pitch ψ, yaw θ, roll φ) at six reference positions in the second trial of an evaluation test before carrying out the study

Target point	*roll (ϕ)*				*yaw (θ)*				*pitch (ψ)*			
	True value (°)	Measurement mean (°)	Measurement SD (°)	Mean to true value (°)	True value (°)	Measurement mean (°)	Measurement SD (°)	Mean to true value (°)	True value (°)	Measurement mean (°)	Measurement SD (°)	Mean to true value (°)
1	0	1.07	1.12	− 1.07	0	− 0.85	0.20	0.85	0	− 0.28	0.36	0.28
2	24	24.88	0.49	− 0.88	0	1.02	0.17	− 1.02	0	0.32	0.27	− 0.32
3	24	26.26	0.29	− 2.26	0	1.49	0.42	− 1.49	90	90.97	1.15	− 0.97
4	24	26.00	0.41	− 2.00	0	− 2.74	0.45	2.74	− 90	− 83.60	0.81	− 6.40
5	24	27.70	0.09	− 3.70	0	0.59	0.27	− 0.59	180	181.61	0.49	− 1.61
6	0	4.40	0.08	− 4.40	0	− 0.35	0.07	0.35	180	182.16	0.90	− 2.16

About 99.7% of the recorded data were within the three-time standard deviation interval around the mean

### Study setup

**Fig. 2:**
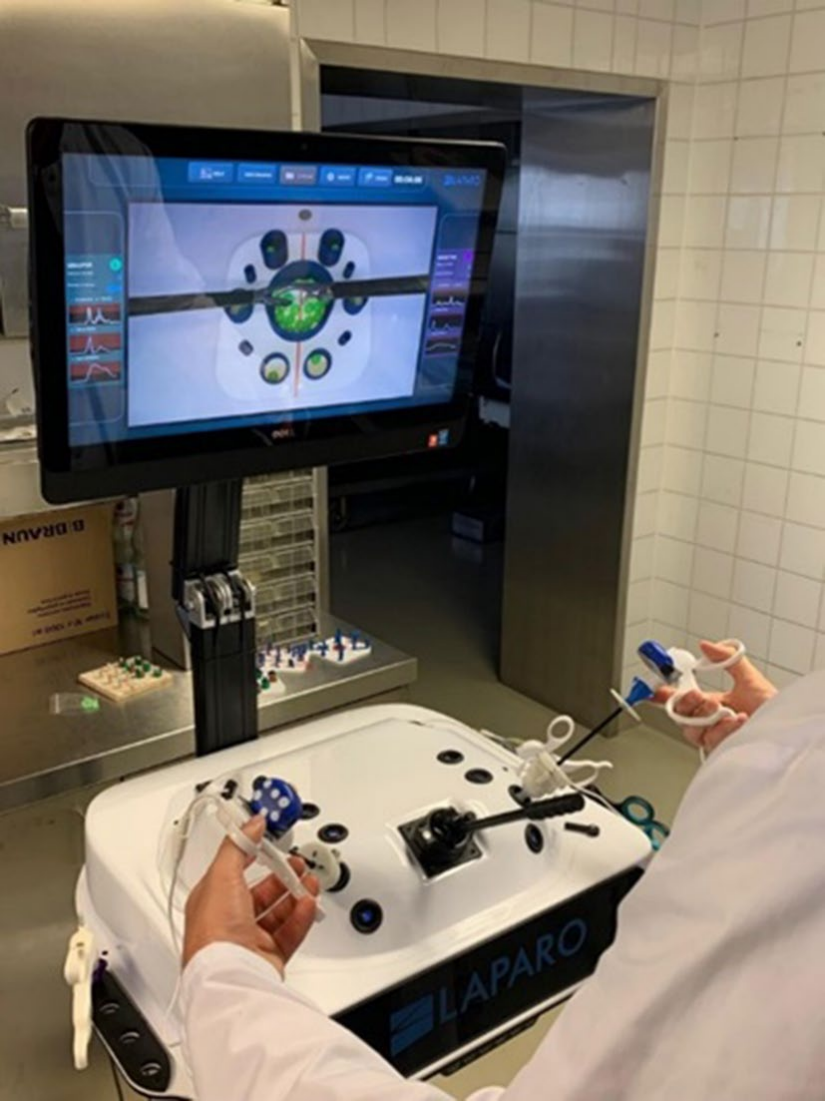
Test setup of laparoscopy trainer. The inertial sensors are attached to the instruments

The study was carried out on the Laparo Analytic (LAPARO Medical Simulators, Wilcza, Poland), consisting of an upper abdomen model. A laparoscopic camera observed the inner space of the model through a static port site and was connected to a viewing screen. The two trocar sites in the model were predefined to make the comparison less variable. A Maryland dissector (LAPARO Medical Simulators, Wilcza, Poland) was placed through the right one, while a laparoscopic grasper (LAPARO Medical Simulators, Wilcza, Poland) passed through the left one. Before beginning, our inertial sensors were calibrated and attached to the handle of the instrument perpendicular to the shaft (Fig. 2).

### Study design

After approval by the ethics committee of the Faculty of Medicine at Ludwig-Maximilians-University Munich, the study was conducted at the Minimally Invasive Surgery Training Lab at the Department of General, Visceral, and Transplant surgery of Ludwig-Maximilians-University Hospital, Munich. Eighteen participants, either medical students or residents, were enrolled in the study. All participants provided written informed consent and agreed to the processing of their data.

Firstly, the participants filled in a questionnaire about demographics and a self-assessment of laparoscopic skills. The study consisted of two tasks; the first task (T_1_) was a peg transfer exercise according to the fundamentals of laparoscopic surgery (FLS) curriculum, while the second task (T_2_) was a ball sorting exercise [13].

**Fig. 3:**
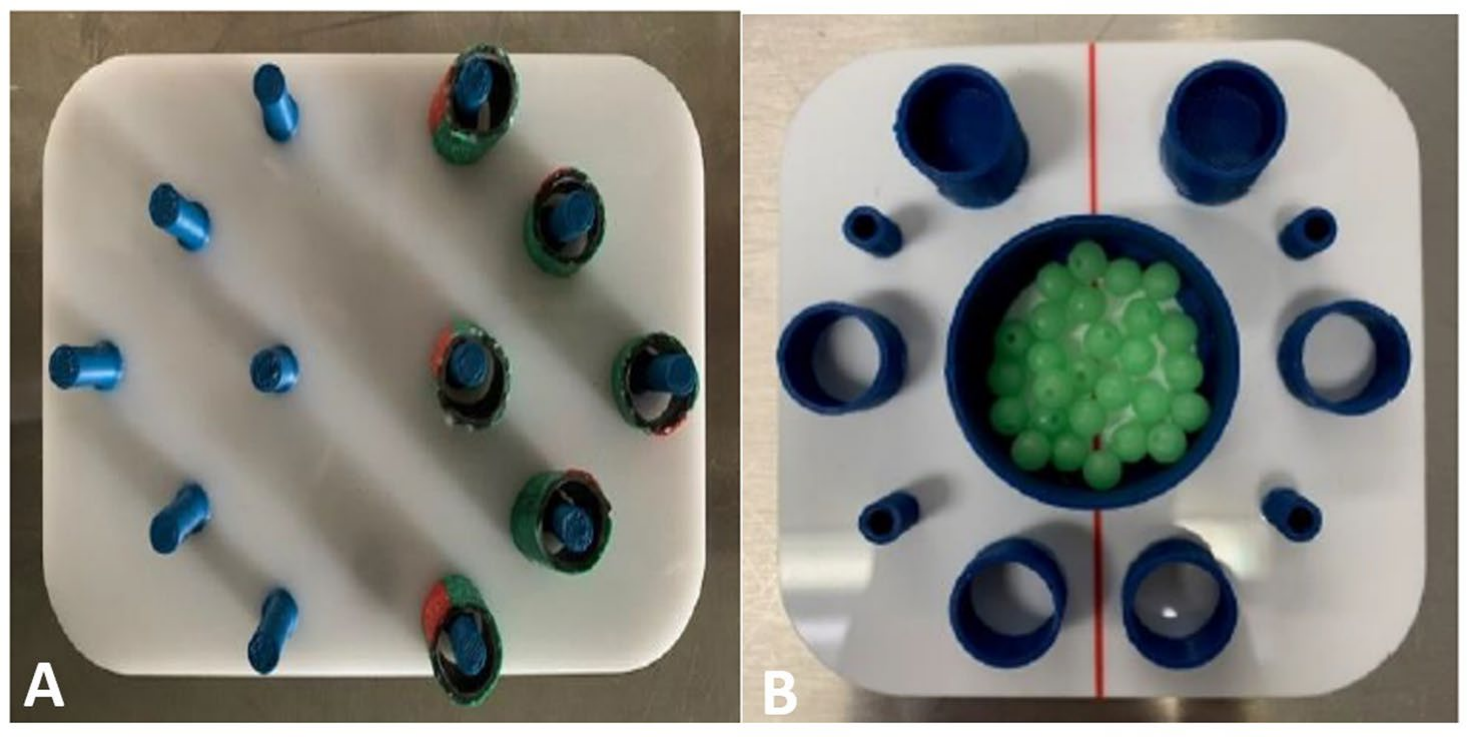
**A** Task 1 (Peg transfer exercise). The task was to move all bodies to the other side, always using the shortest path. The non-dominant hand is used to pick up the bodies, and the dominant hand is used to set them down. **B** Task 2 (Ball sorting exercise) The task was to fill each cylinder with balls with the objective of processing in a clockwise manner starting at 1 o’clock, picking up the balls with the non-dominant hand and dropping them with the dominant hand

In T_1_, we used a board with twelve vertical bars, six bars on the right and the other six on the left, (Fig. 3A). Six tubes were slipped over the bars on one half of the board. The task was to move all objects to the other side, always using the shortest path. The participants were instructed to pick up the objects with the non-dominant hand and set them down with the dominant hand. Dropped objects had to be picked up again with the active instrument.

T_2_ consisted of a container with balls and other small containers around it. The task was to fill each of the smaller containers with balls, clockwise starting at 1 o'clock, picking up the balls with the non-dominant hand and dropping them with the dominant hand, not picking up lost balls (Fig. 3B).

Each participant in the medical student group completed a one-week training according to the FLS curriculum. Each participant performed this evaluation before the laparoscopic training to evaluate their skills. A second evaluation was done at the end of the training period.

### Objective measurements

We selected ten skill parameters for each exercise that were developed based on Hofstad et al. [14]. The parameters were: time (t) "time needed to complete a single task," Perpendicular Angle Sum (pAS) "total angle of the instrument perpendicular to its axis," Standard deviation of perpendicular angles (*σ*_ψ_), Rotatory Angle Sum (rAS) "total angle that the instrument rotated around its axis," Standard deviation of rotatory angle (*σ*_α_), motion smoothness (MS) "total change in acceleration of the instrument," bimanual dexterity (BD) "ability to control two instruments at the same time.", average angular rate (*⍵*) "ratio of angular displacement per time interval," count of swings (CS) "count of moving the instrument back and forth in a time interval" and count of rotations (CR) "count of instrument rotations around its axis."

Pre- and post-training results of the medical students were compared to each other and the results of the physician group. We also compared the results to the Laparo Analytic as a reference to confirm the validity of the developed measuring method.

Also, each medical student's learning success (LS) after training was calculated. We derived LS based on the parameters with significant results as LS = 1/*n* Σ[*P*2/*P*1], where *P*1 represents the magnitude of the parameter before and *P*2 after training. The total *n* describes the number of parameters collected in the experiment. LS, therefore, describes the average relative increase in the other parameters.

### Statistics

For statistical dependence analysis and descriptive statistics, MATLAB R2020b was used, whereas IBM SPSS Statistics 22.0 (SPSS Inc., Chicago, IL) was used for correlation analysis.

To determine the significant difference between the pre-t and post-training results and between the experience levels, the paired t-test and the student’s t-test were appropriate for the normally distributed continuous variables, respectively. The Wilcoxon signed-rank test was appropriate for the skewed continuous variables for pre- and post-training trails and the Mann. Whitney test was used for the different participant groups. We report the values in the format (mean ± standard deviation) when they were normally distributed. *P*-values of *p* < 0.05 were considered significant.

The correlation between the inertial measurement unit (IMU) results in LS_IMU_ and the reference laparo-trainer results LS_LAP_ were visualized using a scatter plot. The strength of the correlation was tested by the Pearson correlation coefficient (*r*).

### Participants

A total of twelve medical students took part in the study. One withdrew for undisclosed reasons. This group (*n* = 11) consisted of two females and nine males. Moreover, six male physicians took part in the study. All participants were right-handed.

Based on a self-assessment questionnaire, the participants' experience levels were classified. While the physicians had advanced experience in dealing with laparoscopic instruments, the medical students were classified into "no previous experience" (6 participants), "only previous ex-vivo experience" (2 participants), and "previous experience as camera holders in the OR" (3 participants).

## Results

### Comparison of medical students’ performance pre-training and post-training (Table 2)

**Table 2:** Detailed results for all upcoming metrics comparing the student’s pre-training and post-training results

Parameter	Exercise 1			Exercise 2		
	Pre-training result	Post-training result	*P* value	Pre-training result	Post-training result	*P* value
	Mean ± SD	Mean ± SD		Mean ± SD	Mean ± SD	
*t* (s)	182.08 ± 113.52	89.76 ± 12.37	**0.011**	150.63 ± 55.12	101.63 ± 30.59	0.1
pAS dominant^a^ (°)	12.40 ± 3.68	14.53 ± 5.41	0.059	10.21 ± 3.89	11.39 ± 4.63	0.168
pAS non-dominant^b^ (°)	11.29 ± 3.79	15.59 ± 5.04	0.106	10.59 ± 5.81	13.55 ± 3.95	0.288
rAS dominant (°)	6.72 ± 3.18	5.57 ± 2.93	**0.027**	5.17 ± 1.81	4.87 ± 2.39	**0.025**
rAS non-dominant (°)	7.10 ± 3.38	5.59 ± 1.46	**0.035**	3.97 ± 0.39	4.05 ± 1.58	**0.046**
MS dominant (m/s^2^)	2.66 ± 3.46	1.44 ± 0.55	0.136	1.52 ± 0.83	1.16 ± 0.56	0.127
MS non-dominant (m/s^2^)	1.51 ± 0.64	1.44 ± 0.59	0.397	1.19 ± 0.54	1.02 ± 0.61	0.245
BD (–)	0.08 ± 0.06	0.09 ± 0.05	0.422	0.06 ± 0.05	0.13 ± 0.03	**0.001**
⍵ dominant (°/s)	12.23 ± 2.54	9.46 ± 1.94	**0.004**	10.14 ± 2.60	7.41 ± 1.95	**0.006**
*⍵* non-dominant (°/s)	11.94 ± 4.51	10.20 ± 2.11	0.132	8.29 ± 1.30	7.50 ± 2.07	0.149
CS dominant (–)	348 ± 210.82	117.36 ± 31.64	**0.002**	208.27 ± 101.99	98.82 ± 58.99	**0.004**
CS non-dominant (–)	352.45 ± 302.68	124.36 ± 32.46	**0.016**	210.73 ± 88.06	111.82 ± 64.95	**0.004**
CR^h^ dominant (–)	240.18 ± 163.52	98.64 ± 24.68	**0.008**	149.55 ± 72.33	90.18 ± 59.32	**0.024**
CR non-dominant (–)	282.55 ± 256.73	107.73 ± 38.72	**0.024**	173.73 ± 86.33	104.36 ± 64.39	**0.023**

Bold values indicate statistical significance (*p* < 0.05)

All the continuous variables are given in the Mean, Standard Deviation (SD) and *p* value

*t* time, *pAS* Perpendicular angle sum, *rAS* Rotary angle sum, *MS* Motion smoothness, *BD* bimanual dexterity, *⍵* Average angular rate, *CS* count of swings, *CR* count of rotations

^a^Dominant the dominant hand

^b^Non-dominant the non-dominant hand

The time [sec] on task for T_1_ shortened from 182.08 s ± 113.52 s to 89.76 s ± 12.37 s after the training and for T_2_ from 150.63 s ± 55.12 s to 101.63 s ± 30.5 s. The difference in T_1_ was significant (*p* = 0.011).

The rotary angle sum [°] reduced significantly in T_1_ for both the dominant hand (from 6.72° ± 3.18° to 5.57° ± 2.93°, *p* = 0.027) and the non-dominant hand (from 7.1° ± 3.38° to 5.59° ± 1.46°, *p* = 0.035). In T_2_, we also observed a significant reduction for both the dominant hand (from 5.17° ± 1.81° to 4.87° ± 2.39°, *p* = 0.025) and the non-dominant hand (from 3.9° ± 0.39° to 4.05° ± 1.58°, *p* = 0.046).

The count of swings significantly reduced in T_1_ for the dominant hand (from 348.00 ± 210.82 to 117.36 ± 31.64, *p* = 0.002) and the non-dominant hand (from 352.45 ± 302.68 to 124.36 ± 32.46, *p* = 0.016) as well as in T_2_ for both the dominant hand (from 208.27 ± 101.99 to 98.82 ± 58.99, *p* = 0.004) and the non-dominant hand (from 210.73 ± 88.06 to 111.82 ± 64.95, *p* = 0.004).

Count of rotations showed significant reductions in T_1_ for both the dominant (from 240.18 ± 163.52 to 98.64 ± 24.68, *p* = 0.008) and the non-dominant hand (from 282.55 ± 256.73 to 107.73 ± 38.72, *p* = 0.024). In T_2_, we saw similar results for the dominant hand (from 149.55 ± 72.33 to 90.18 ± 59.32, *p* = 0.024) and the non-dominant hand (from 173.73 ± 86.33 to 104.36 ± 64.39, *p* = 0.023).

The results of bimanual dexterity showed significant differences only for T_2_ (from 0.06 ± 0.05 to 0.13 ± 0.03, *p* = 0.001)(for T_1_ see Table 2).

The average angular rate (°/s) showed a significant reduction for only the dominant hand in both T_1_ (from 12.23 ± 2.54 to 9.46 ± 1.94, *p* = 0.004) and T_2_ (from 10.14 ± 2.60 to 7.41 ± 1.95, *p* = 0.006).

For motion smoothness, we measured improvements for T_1_ and T_2_, as well as for the average angular rate for the non-dominant hand, although the differences were not significant (see Table 2).

### Comparison of medical students’ performance and physicians’ performance (Table 3)

**Table 3:** Detailed results for all upcoming metrics comparing the students’ and physicians’ results

Parameter	Exercise 1			Exercise 2		
	Pre-training result (Medical students)	Exercise result (Physicians)	*P* value	Pre-training result (Medical students)	Exercise result (Physicians)	*P* value
	Mean ± SD	Mean ± SD		Mean ± SD	Mean ± SD	
*t* (s)	182.08 ± 113.52	112.92 ± 30.99	**0.041**	150.63 ± 55.12	88.30 ± 18.82	**0.022**
pAS dominant^a^ (°)	12.40 ± 3.68	109.30 ± 32.94	0.070	10.21 ± 3.89	82.27 ± 37.68	0.54
pAS non-dominant^b^ (°)	11.29 ± 3.79	115.00 ± 48.83	0.209	10.59 ± 5.81	83.76 ± 33.16	0.088
rAS dominant (°)	6.72 ± 114.19	5.57 ± 53.74	0.33	5.17 ± 1.81	83.02 ± 35.48	0.278
rAS non-dominant (°)	7.10 ± 100.35	5.59 ± 132.91	0.680	3.97 ± 0.39	82.97 ± 30.62	0.371
MS dominant (m/s^2^)	2.66 ± 3.46	1.55 ± 0.61	0.160	1.52 ± 0.83	1.22 ± 0.79	0.239
MS non-dominant (m/s^2^)	1.51 ± 0.64	1.42 ± 0.49	0.378	1.19 ± 0.54	1.36 ± 0.53	0.874
BD (–)	0.08 ± 0.06	0.15 ± 0.13	0.136	0.06 ± 0.05	0.27 ± 0.36	0.184
*⍵* dominant (°/s)	12.23 ± 2.54	11.41 ± 3.06	0.274	10.14 ± 2.60	9.28 ± 2.65	0.266
*⍵* non-dominant (°/s)	11.94 ± 4.51	10.68 ± 2.29	0.228	8.29 ± 1.30	8.82 ± 2.07	0.882
CS dominant (–)	348 ± 210.82	166.5 ± 79.62	**0.012**	208.27 ± 101.99	97.33 ± 34.01	**0.003**
CS non-dominant (–)	352.45 ± 302.68	147.00 ± 49.21	**0.025**	210.73 ± 88.06	103.50 ± 18.96	**0.001**
CR dominant (–)	240.18 ± 163.52	144.00 ± 64.11	0.053	149.55 ± 72.33	83.00 ± 36.14	**0.012**
CR non-dominant (–)	282.55 ± 256.73	127.83 ± 68.12	**0.042**	173.73 ± 86.33	92.33 ± 34.62	**0.008**

Bold values indicate statistical significance (*p* < 0.05)

All the continuous variables are given in the Mean, Standard Deviation (SD) and *p*-value

*t* time, *pAS* perpendicular angle sum, *rAS* rotary angle sum, *MS* Motion smoothness, *BD* Bimanual dexterity, *⍵* Average angular rate, *CS* Count of swings, CR Count of rotations

^a^Dominant the dominant hand

^b^Non-dominant the non-dominant hand

In the comparison between the medical student’s and the physician’s pre-training trial, there was a significant difference in time in both T_1_ (182.08 s ± 113.52 s vs. 112.92 s ± 30.99 s, *p* = 0.041) and T_2_ (150.63 s ± 55.12 s vs. 88.30 s ± 18.82 s, *p* = 0.022).

The count of swings in T_1_ was significantly smaller for the physicians in both the dominant hand (348.00 ± 210.82 vs. 166.5 ± 79.62, *p* = 0.012) and the non-dominant hand (352.45 ± 302.68 vs. 147.00 ± 49.21, *p* = 0.025). The same applies in T_2_ for the dominant hand (208.27 ± 101.99 vs. 97.33 ± 34.01, *p* = 0.003) and the non-dominant hand (210.73 ± 88.06 vs. 103.50 ± 18.96, *p* = 0.001).

The count of rotation also showed significantly smaller values for the non-dominant hand in the physician sub-group (282.55 ± 256.73 vs. 127.83 ± 68.12, p = 0.042). In T_2_, we measured significantly smaller values in the physician sub-group for both the dominant hand (149.55 ± 72.33 vs. 83.00 ± 36.141, p = 0.012) and the non-dominant hand (from 173.73 ± 86.33 to 92.33 ± 34.62, *p* = 0.008).

In contrast, we found no significant differences for any parameter comparing the post-training trials of the medical students against the physicians’ trials (Table 4).

**Table 4:** Post-training results of medical students versus physician’s results

Parameter	Exercise 1			Exercise 2		
	Post-training result (Medical students)	Exercise result (Physicians)	*P* value	Post-training result (Medical students)	Exercise result (Physicians)	*P* value
	Mean ± SD	Mean ± SD		Mean ± SD	Mean ± SD	
*t* (s)	89.76 ± 12.37	112.92 ± 30.99	0.935	101.63 ± 30.59	88.30 ± 18.82	0.142
pAS dominant^a^ (°)	14.53 ± 5.41	109.30 ± 32.94	0.618	11.39 ± 4.63	82.27 ± 37.68	0.120
pAS non-dominant^b^ (°)	15.59 ± 5.04	115.00 ± 48.83	0.833	13.55 ± 3.95	83.76 ± 33.16	0.104
rAS dominant (°)	5.57 ± 2.93	5.57 ± 53.74	0.984	4.87 ± 2.39	83.02 ± 35.48	0.866
rAS non-dominant (°)	5.59 ± 1.46	5.59 ± 132.91	0.892	4.05 ± 1.58	82.97 ± 30.62	0.901
MS dominant (m/s^2^)	1.44 ± 0.55	1.55 ± 0.61	0.637	1.16 ± 0.56	1.22 ± 0.79	0.560
MS non-dominant (m/s^2^)	1.44 ± 0.59	1.42 ± 0.49	0.473	1.02 ± 0.61	1.36 ± 0.53	0.874
BD (–)	0.09 ± 0.05	0.15 ± 0.13	0.151	0.13 ± 0.03	0.27 ± 0.36	0.184
*⍵* dominant hand (°/s)	9.46 ± 1.94	11.41 ± 3.06	0.900	7.41 ± 1.95	9.28 ± 2.65	0.917
*⍵* dominant (°/s)	10.20 ± 2.11	10.68 ± 2.29	0.661	7.50 ± 2.07	8.82 ± 2.07	0.882
CS dominant (–)	117.36 ± 31.64	166.5 ± 79.62	0.901	98.82 ± 58.99	97.33 ± 34.01	0.474
CS non-dominant (–)	124.36 ± 32.46	147.00 ± 49.21	0.829	111.82 ± 64.95	103.50 ± 18.96	0.350
CR dominant (–)	98.64 ± 24.68	144.00 ± 64.11	0.926	90.18 ± 59.32	83.00 ± 36.14	0.381
CR non-dominant (–)	107.73 ± 38.72	127.83 ± 68.12	0.737	104.36 ± 64.39	92.33 ± 34.62	0.312

All the continuous variables are given in the Mean, Standard Deviation (SD) and *p* value

*t* time, *pAS* Perpendicular angle sum, *rAS* Rotary angle sum, *MS* motion smoothness, *BD* bimanual dexterity, *⍵* average angular rate, *CS* count of swings, *CR* count of rotations

^a^Dominant the dominant hand

^b^Non-dominant the non-dominant hand

**Fig. 4:**
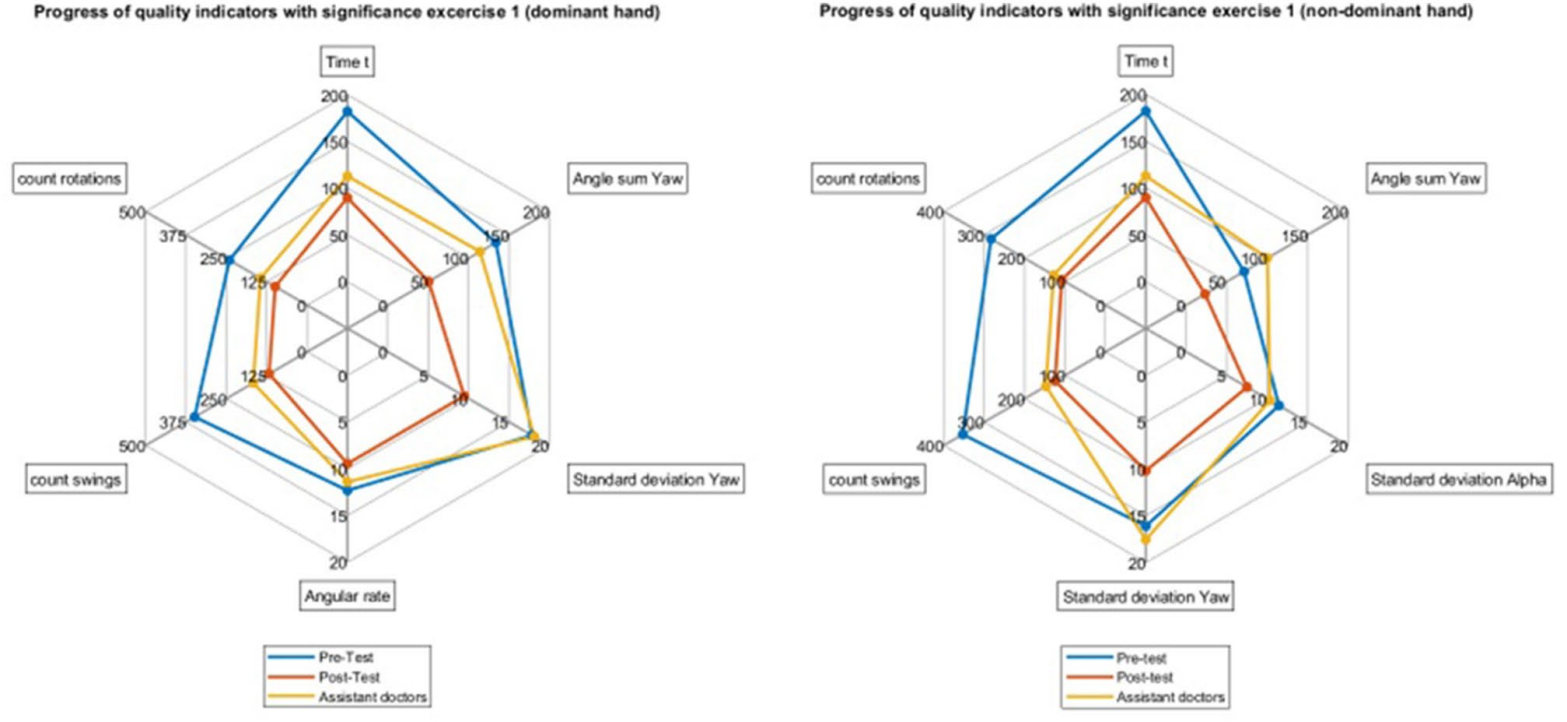
Spider plots for the mean values for the pre- and post-training results of the medical students (left) and the results of the physician (right) for the significant parameters in T_1_

**Fig. 5:**
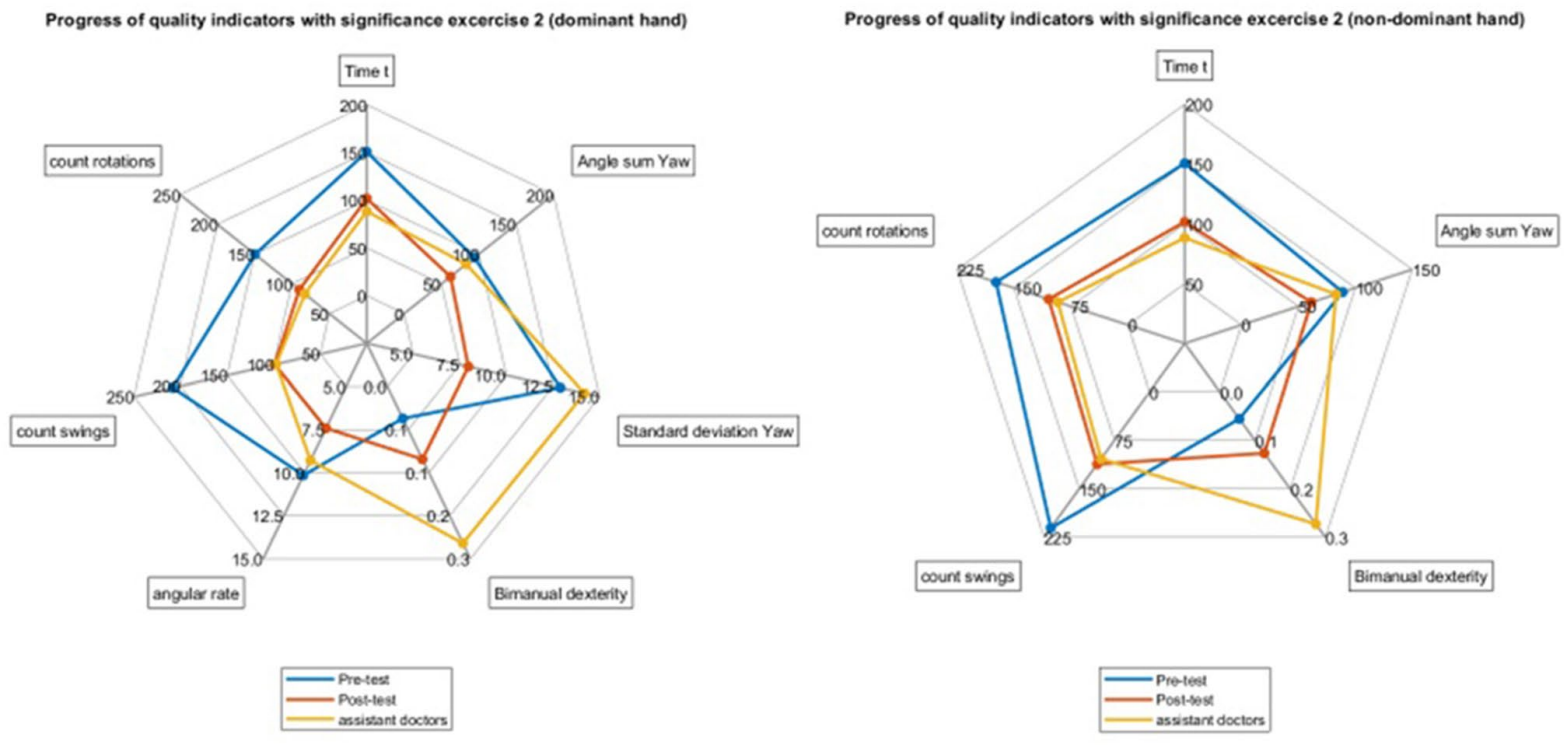
Spider plots for the mean values for the pre- and post-training results of the medical students (left) and the results of the physicians (right) for the significant parameters in T_2_

All comparisons between the mean values of students' pre- and post-training results and the physicians for all significant parameters are visualized as spider plots in (Figs. 4 and 5).

**Fig. 6:**
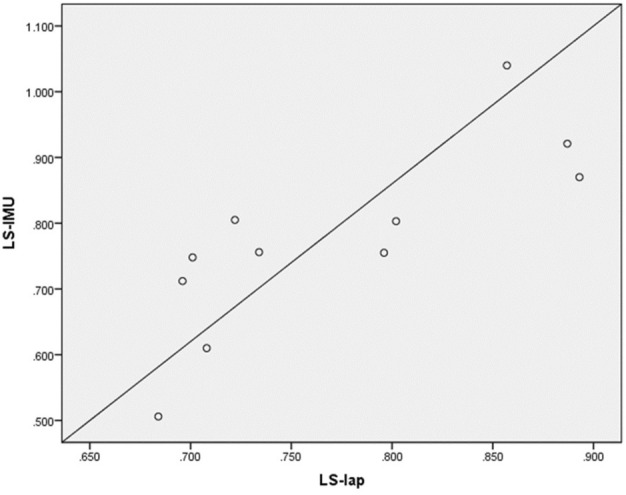
Scatter plot diagram of learning success. It showed a positive correlation between LS_lap_ and LS_IMU_ values for the eleven medical students. LS_lap_ = Learning success Laparo-Trainer, LS_IMU_ = Learning success inertial measurement unit. Pearson’s *r* = 0.79, *p* = 0.003

Regarding the learning success of each medical student, we found a strong positive correlation between the measurements obtained from the inertial sensor LS_IMU_ and those obtained from the trainer LS_Lap_ with Pearson’s *r* = 0.79 with *p* = 0.003 (Fig. 6).

## Discussion

In the current, study we developed a laparoscopic instrument motion-tracking method based purely on inertial sensors to evaluate the learning success of surgeons besides instrument motion tracking. Our results show an overall improvement in the measured fine motor parameters as well as the time it took medical students to complete their tasks. These results indicate the effectiveness of the training course and the possible success of motion tracking with the inertial sensors used in our study, which were consistent with similar training simulator results [15, 16].

Although all the parameters appear abstract, they are linked with the participant’s performance in completing the tasks. Three parameters emerged that objectified improved performance: The rotary angle sum (total movement of the instruments around their axis), the count of swings (number of forward and backward movements of the instruments in a time interval) and the count of rotation (number of instrument rotations around their axis). A reduction in these parameters can each be interpreted as an increased efficiency of instrument handling. This could, when transferred to a real surgical situation, give an indication of a more targeted preparation. On the other hand, these parameters show that incorrect or unnecessary movements are avoided, which may otherwise lead to unnecessary injuries or complications during surgery. Thus, these parameters could serve as an indication of the safety of the surgical procedure. This needs to be investigated in further studies with tasks of higher difficulty or in surgery simulations. Surgical dexterity is usually reflected through the time needed to perform some surgical manipulation and constantly improves with ongoing training. Munz et al. indicated that task-related time was not significantly improved as the participant tried to be more cautious and not rush to accomplish their tasks [17]. In contrast, the time needed to accomplish T_1_ and T_2_ for our student subgroup was significantly reduced after training. This can be clarified by the efficacy of the illustrated training course, the participants' familiarity with the trainer's fixed criteria, and the successful acquisition of essential psychomotor coordination skills. On the other hand, the post-training times for T_1_ and T_2_ were not significantly different compared to the physicians, indicating the ability to transfer the developed skills into real situations in ex-vivo laparoscopic training.

Motion smoothness showed only some improvements, especially for the dominant hand in the medical students in T_1_ and T_2_. Therefore, this was consistent with the detected improvement in the novice participants of the study of Hiemstra et al. [18]. These results reflect the lack of experience of the medical students, which was relatively improved after the training course. Moreover, our results regarding motion smoothness showed no significant difference compared to the physicians, parallel to the study results of van Empel et al. with another box trainer [19]. This is consistent with Sanchez et al., who could not show the efficacy of a computer-based algorithm in classifying the users of an augmented reality-based trainer into experienced and non-experienced based on their learning curve [20]. Therefore, the absolute figures resulting from our results should not be considered an accurate indicator to differentiate between experienced laparoscopic users and novices.

On the other hand, Botden et al. measured a significant difference in motion smoothness between experts and novices in suturing on the ProMIS laparoscopic hybrid box trainer. This can be attributed to the different platform and the fact that suturing techniques in this study require more technical skills where the difference between experts and amateurs is more obvious [21].

Bimanual dexterity showed significant improvement only for T_2_, while in T_1_, there was no improvement. This can be explained by the lack of experience of the medical students, and we expect that this skill will improve clearly with regular exercise.

Our study revealed no significant difference in the parameter BD between the medical students and physicians. However, based only on motion- parameter analysis, we cannot conclude that they genuinely represent quality indicators for surgical skills. In contrast, classifying the participant’s skills into novice and professionals was achieved with 100% accuracy in the study of Horeman et al., who have applied combined force and motion-based parameters by 3DOF force sensor on a more adjusted experimental task mimicking the in-vivo environment (Endopath EXCEL, Johnson & Johnson) [2]. Moreover, careful adjustment between task’s characteristic (e.g., elasticity and friction) and training goal should be secured, in order to avoid unpredictable fluctuations in the individual learning curve of both novices and professionals [3].

When examining the differences between the students pre-training against the physicians, we found several statistical significances in performance. In contrast, we found none in the students post-training against the physicians. While we did not perform a statistical test for equivalence of the student's performance after training and the physicians, studying the spider plots, we observed, at least for our participants, that the students seem on par, if not slightly better at the tasks than the physicians. The grain of salt here is that the students were already familiar with the setup and the tasks when they repeated the experiment, and the physicians were not. We do not know how good of a simulation of actual laparoscopic surgery the tasks were. On the other hand, this underlines the high value of laparoscopic training.

The study by Pagador et al. has applied tool motion analysis to evaluate the surgical skills for different subtasks of the laparoscopic suturing procedure. However, they suggested that more modifications are needed to create a complete link between decomposed motion analysis and overall performance [22]. On the other hand, our current study has accurately monitored the learning success of medical students after the training through statistically significant pre-set parameters. The learning success gained from the sensor (LS_IMU_) was compared to the reference learning success measured by the commercial trainer (LS_Lap_), where a strong correlation was found.

Mentioning other training models, Horeman et al. developed a new box trainer for quantitative measurement of task time, force and motion data for single and multiple port laparoscopy [10]. Some of the sensors were included inside and at the top of the box. Although we were not able to measure forces, the advantage of our system is that it is possible to include it in any possible training systems, because it is only attached to the handle of the instrument, and individual from other systems. Beside that we might be able to use it in a sterile setting in the OR using a sterile cover. We might also be able to use it in multiple as well as single port surgery without the need of a completely new trainings system.

Discussing possible instrument tracking possibilities, the inertial sensor was able to transmit the motion data of the adherent instrument also outside the field of view. This could be a clear advantage over the analysis of skill development based only on software programs, as in computer vision tracking [23]. Hence, it could not only be used to track the learning curve of the surgeons, but also it would help learn from the essential skills of the advanced surgeons. In addition to recording, examining, and comparing the surgeon's movement, it also offers the opportunity to compare surgical approaches on a patient-specific basis and gain further insight using motion analysis.

Since the sensors can be easily integrated into the surgical workflow without any occlusion problems, they could constantly assess the performance of laparoscopic surgeons in real procedures in patients. As the current study developed a sterile cover for the heat-sensitive sensors, an upcoming study should apply the developed inertial sensor system in the OR to collect structured data for various standard procedures. In future for example, each surgeons’ experiences could be archived and subsequently used for digital assistance systems [24, 25].

Seifert et al. stated that position determination based only on microelectromechanical inertial sensors is not suitable regarding the current state of the sensors [26]. They assumed that even if the inertial sensor was optimally calibrated, there was too much scattering and systematic deviation. In our trial, the deviation of the measured value from the natural state was limited, particularly by suitable mathematical models such as the Kalman-filter. Moreover, the absolute value of the orientation at a specific time was not directly relevant to our intended study. Hence, skill assessment occurred over time intervals where the sum of deviation approached zero. However, the low sensor accuracy, even with the Kalman-filter should be improved. E.g., by fusion of the sensor data with an additional external sensor to have a more stable reference value for fixation in space, which we did not implement due to increased cost.

For completeness and as a limitation of our current study, although we have shown that some motion parameters can be perfectly derived from inertial sensors, other parameters such as depth perception and path length were not investigated. These two, among others, while at least as relevant for transfer from simulation to real operations, require other tracking tools.

Secondly, the initial starting point of the measurements had to be specified in the study setup and could not be determined by the sensor itself. This does not seem to be relevant for this pilot study, since all tasks were performed on one and the same model, but must be determined and revised as a limitation for testing during a real operation.

Also, to differentiate well between professional surgeons and novices we should consider more complex tasks to implement with a more accurate determination of instrument orientation. In this regard, we could provide an excellent opportunity to monitor learning progress in laparoscopic surgery, but the low number of participants still limits this. This should be re-evaluated on a larger cohort.

## Conclusion

The current study showed a good and valid performance of inertial measurement units as a possible tool for instrument tracking and surgical skill assessment. We demonstrated that in an ex-vivo environment, the learning progress of laparoscopic surgery trainees can be monitored. With this cost-effective tool, we examined fine motor parameters, which are able to objectify improvements in instrument handling. With the possibility of sterile use inside the operation room, it might also provide the opportunity to monitor and improve one's skills and abilities in a real environment.
